# Exploring the Validity of an Optoelectronic Integrated Cone Beam Computed Tomography Jaw Tracking System

**DOI:** 10.3390/jcm12124145

**Published:** 2023-06-20

**Authors:** Hayo C. van der Helm, Arjan J. A. Dieters, Pieter U. Dijkstra, Wicher J. van der Meer, Anne Marie Kuijpers-Jagtman

**Affiliations:** 1 Department of Orthodontics, University Medical Center Groningen, University of Groningen, Hanzeplein 1, 9713 GZ Groningen, The Netherlandsa.m.kuijpers-jagtman@umcg.nl (A.M.K.-J.); 2 Department of Dentistry and Oral Hygiene, University Medical Center Groningen, University of Groningen, Hanzeplein 1, 9713 GZ Groningen, The Netherlands; 3 Department of Rehabilitation, University Medical Center Groningen, University of Groningen, Hanzeplein 1, 9713 GZ Groningen, The Netherlands; 4 Department of Oral and Maxillofacial Surgery, University Medical Center Groningen, University of Groningen, Hanzeplein 1, 9713 GZ Groningen, The Netherlands; 5 Sirindhorn School of Prosthetics and Orthotics, Faculty of Medicine Siriraj Hospital, Mahidol University, 14 Arun Amarin Rd, Bangkok 10700, Thailand; 6 Department of Orthodontics and Dentofacial Orthopedics, School of Dental Medicine, Medical Faculty, University of Bern, Freiburgstrasse 7, CH-3010 Bern, Switzerland; 7 Faculty of Dentistry, Universitas Indonesia, Campus Salemba, Jalan Salemba Raya No. 4, Jakarta 10430, Indonesia

**Keywords:** jaw motion tracking, 4D, cone beam computed tomography (CBCT), validation, mandible, optoelectronic

## Abstract

Jaw motion tracking functionalities of cone beam computed tomography (CBCT)-scanners can visualize, record, and analyze movements of the mandible. In this explorative study, the validity of the 4D-Jaw Motion module (4D-JM) of the ProMax 3D Mid CBCT scanner (Planmeca, Helsinki, Finland) was tested in vitro. The validity of the 4D-JM was accepted if values differed less than 0.6 mm (three voxels sizes) from the gold standard. Three dry human skulls were used. CBCT scans, the gold standard, were taken in eight jaw positions and exported as three-dimensional (3D) models. Individualized 3D-printed dental wafers ensured the correct positioning of the mandible. Jaw positions were recorded with the 4D-JM tracking device and exported as 3D models. The coordinates of six reference points for both superimposed 3D models were obtained. The differences in the *x*, *y* and *z*-axis and the corresponding vector differences between gold standard 3D models and 4D-JM models were calculated. For the mandible 10% and for the maxilla 90% of the vector differences fell within 0.6 mm of the gold standard. With an increasing vertical jaw opening, larger differences between the gold standard and the 4D-JM 3D models were found. The smallest differences of the mandible were observed on the *x* axis. In this study, the 4D-JM validity was not acceptable by the authors’ predefined standards.

## 1. Introduction

Jaw motion tracking functionalities of cone beam computed tomography (CBCT)-scanners enable the visualization and quantitative analysis of movements of the temporomandibular joints (TMJs) [1,2].

In 2014, Dentsply Sirona released the jaw motion tracker functionality software SICAT JMT+ (Version BT, Dentsply Sirona Inc., Charlotte, NC, USA), which uses the propagation times of acoustic signals for jaw motion tracking. The accuracy of that system was explored in a case study [3]. The authors stated the SICAT JMT+ to be accurate and reliable, but further studies were needed [3]. The SICAT JMT+ has been applied for measuring jaw movements after total joint replacement and after orthognathic surgery [4,5].

In 2016, Planmeca released the 4D Jaw Motion module (4D-JM) for the ProMax 3D Mid and Max CBCT scanners (Version 30004987, Planmeca, Helsinki, Finland). The CBCT scanner with the 4D-JM is an optoelectronic tracking device with two integrated digital cameras able to track reflecting spheres, which are temporarily attached to the cranium and mandible. Real-time motion of the TMJs can be visualized in the software and measured. The 4D-JM has been applied to determine the patient-specific rotation center of the condylar head during mouth opening for total temporomandibular joint replacements [6].

However, no research is available that reports the validity of the jaw motion tracking functionalities of CBCT scanners and their corresponding software. Therefore, we explored the validity of the 4D-JM for different jaw positions in dry human skulls. We hypothesized that the 4D-JM could accurately track different jaw positions within 0.6 mm.

## 2. Materials and Methods

### 2.1. Research Design

This in vitro study was performed at the University Medical Center Groningen (UMCG) at the Department of Orthodontics from March 2022 to September 2022. The Institutional Review Board (METc UMCG) confirmed that this research was not a clinical research with human subjects as meant in the Medical Research Involving Human Subjects Act (WMO) and, therefore, ethical approval was not needed (METc 2021/652).

Three adult dry skulls were selected. Inclusion criteria were more than 20 teeth present, possibility to position the skull on the positioning table and to place the mandible in different vertical, lateral and protruded positions.

### 2.2. Preparation of the Skull and Wafers

The dentition of each dry skull was scanned using an intra-oral scanner (True definition, 3M, Saint Paul, MN, USA) and a CBCT scan with a high-resolution protocol (settings of 90 kV voltage, 10 mA dose, 36 s exposure time, voxel size 0.2 mm) of each skull was taken with the ProMax 3D Mid CBCT scanner. The intra-oral scan and the CBCT scans were superimposed into 3D models. Eight wafers per skull were digitally designed using these 3D models in Meshmixer (Autodesk, San Rafael, CA, USA) to provide the vertical, lateral and protruded positions of the mandible needed for the measurements (Right 5 mm, Left 5 mm, Protrusion 5 mm, Protrusion 10 mm, Occlusion, Open 10 mm, Open 30 mm and Open 50 mm). The wafers were 3D-printed using the Sigma R19 3D-printer (BCN3D Technologies, Barcelona, Spain) with polylactic acid (PLA) filament, diameter 2.85 mm (BCN3D Technologies, Barcelona, Spain). The wafers were designed to position the mandibular central incisors in the exact positions relative to the maxillary central incisors. The wafers designed for horizontal positioning ensured an adequate vertical separation.

**Figure 1:**
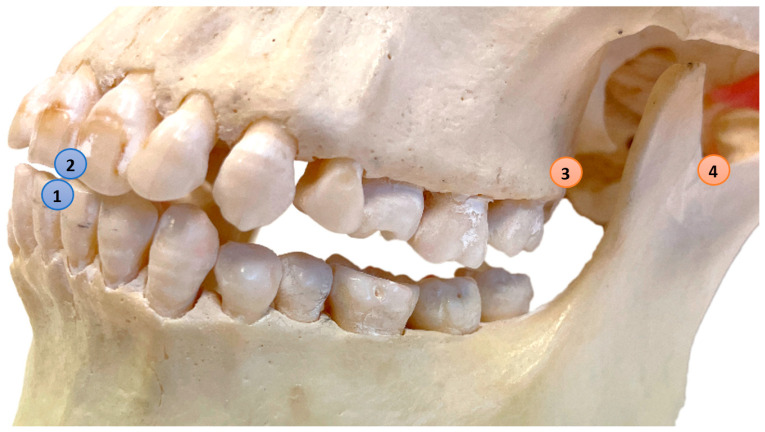
Reference points: (1) Mandible–Central: Most mesial point of the incisal edge of the lower right central incisor; (2) Maxilla–Central: Most mesial point of the incisal edge of the upper right central incisor; (3) Maxilla–Left: Most dorsal caudal point of the left maxillary tuberosity (marker), Maxilla–Right is located on the contralateral side (marker); (4) Mandible–Left: Most caudal point in the left mandibular notch (marker), Mandible–Right is located on the contralateral side (marker).

**Figure 2:**
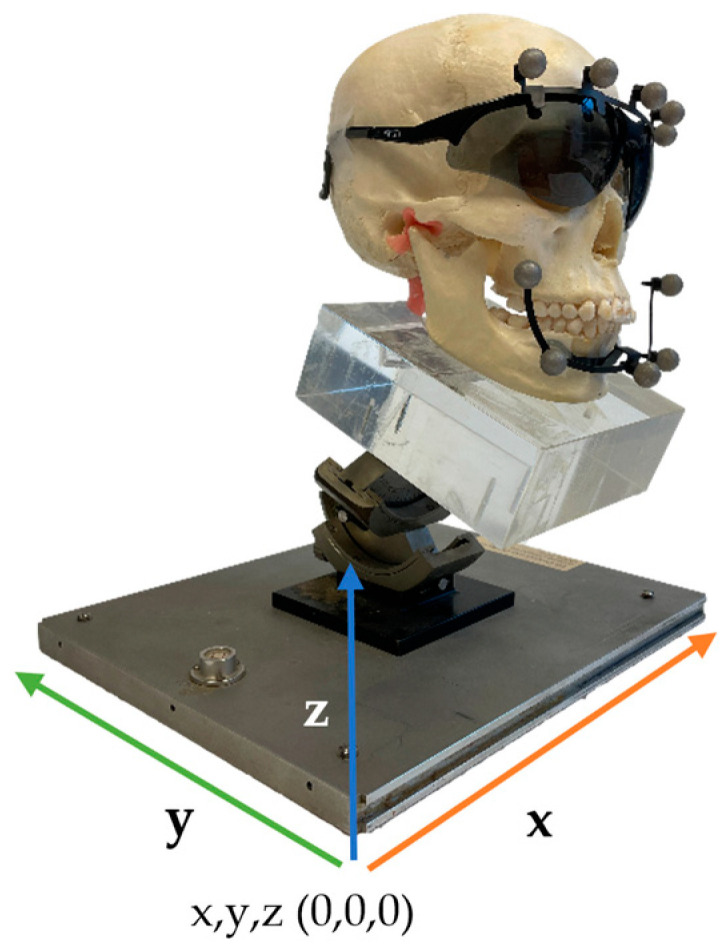
Set-up of the skull with the 4D-JM tracking device on the positioning table.

Four custom made radio-opaque markers with a diameter of 0.5 mm were attached to specific points on the skull’s cranium and mandible to serve as reference points. Two additional anatomical landmarks were chosen; the most mesial point of the incisal edge of the maxillary and mandibular right central incisor (Figure 1). The 4D-JM tracking device (Planmeca, Helsinki, Finland), equipped with reflective spheres, was affixed to the skull using non-reflective matte adhesive tape (Office Depot, Venlo, The Netherlands) (Figure 2). These reflective spheres were tracked by the integrated cameras of the CBCT scanner. Since the height of the articular disk is approximately 2 mm [7], the articular disk was simulated by means of 2 mm radiolucent utility wax (Set Up Regular, Cavex, Haarlem, The Netherlands).

### 2.3. Data Collection

**Figure 3:**
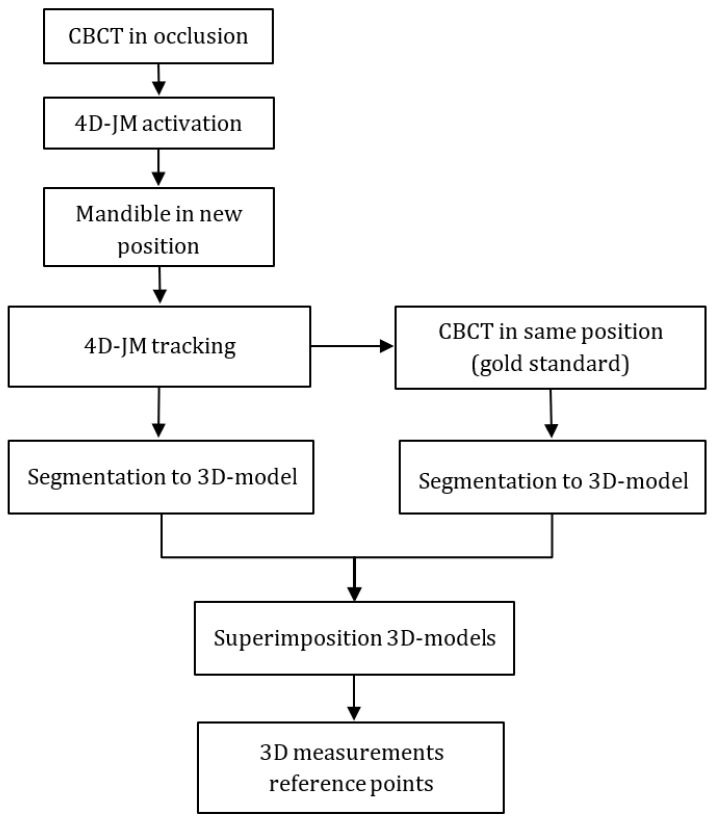
Measurement protocol.

The skulls were positioned in the CBCT scanner with the aid of a positioning table (Figure 2). Data collection started with a CBCT scan with a high-resolution protocol (settings of 90 kV voltage, 10 mA dose, 36 s exposure time, voxel size 0.2 mm) with the dentition in occlusion. The 4D-JM module was activated in the Romexis software (Version 6.2.1.19 2-9-21, Planmeca, Helsinki, Finland) and the most recent protocol (Number 30004987 Revision 7) of the manufacturer was followed. Within this module the 4D-JM reflective spheres were digitally selected to enable camera tracking. This allowed real-time tracking of the 3D mandibular movement without the need for additional ionizing radiation. Subsequently, the mandible was placed into the desired position using the custom 3D-printed wafers and repositioned on the positioning table. The cameras recorded the new position of the mandible with the reflective spheres, and the mandible was virtually placed in the new position within the 4D-JM software (Version 6.2.1.19 2-9-21). A 3D model was then segmented in this virtual position with the lowest segmentation threshold possible to separate the mandible and maxilla into two distinct 3D models. Without moving the skull, a CBCT scan with high-resolution protocol was taken in this new position and segmented to serve as the gold standard 3D model (Figure 3).

The gold standard and 4D-JM 3D models were superimposed onto the three maxillary reference points (Figure 1). The superimpositions were conducted using Geomagic Control X software (Version 2022.1.0, 3D Systems, Morrisville, NC, USA) with a maximum tolerance of two voxel sizes (0.4 mm). Achieving a perfect superimposition was not feasible due to the different segmentation methods of the gold standard CBCT and the 4D-JM. The 4D-JM utilizes a fixed region growing method, which yields a slightly different 3D model than the thresholding method used in the gold standard CBCT.

The coordinates of six reference points in both the gold standard and 4D-JM 3D models were obtained. To explore the validity of the 4D-JM, measurements were performed by the first author (HH). To determine inter- and intra-observer reliability the coordinates of the six reference points were measured twice by two observers (HH and AD) with a two-week interval.

### 2.4. Data Exploration

Data were analyzed in IBM SPSS Statistics version 28 (IBM Corp., Armonk, NY, USA). Intra- and inter-observer reliability were assessed using an intraclass correlation coefficient (ICC, two-way mixed effects model, absolute agreement, single measures).

The differences on the *x*, *y* and *z* axis as well as the corresponding vector difference, between the six reference points of the gold standard 3D models and the 4D-JM models were calculated in Geomagic Control X software (Version 2022.1.0). The primary outcome variable, the vector difference between gold standard and the 4D-JM, was calculated as follows:

Vector difference = √((X_gs_ − X_4D-JM_)^2^ + (Y_gs_ − Y_4D-JM_)^2^ + (Z_gs_ − Z_4D-JM_)^2^)

X_gs_ = x-coordinate gold standard; X_4D-JM_ = x-coordinate 4D-JM; Y_gs_ = y-coordinate gold standard; Y_4D-JM_ = y-coordinate 4D-JM; Z_gs_ = z-coordinate gold standard; Z_4D-JM_ = z-coordinate 4D-JM.

Thereafter, the percentage vector differences less than 0.6 mm (=3 voxel sizes), 1.2, 1.8 and 2.4 mm were calculated for mandible and maxilla. A one sample t-test was performed to analyze the mean difference of the maxilla vector differences compared to zero because zero would indicate a perfect superimposition.

Differences between the gold standard and the 4D-JM in the *x* axis, *y* axis, and *z* axis were calculated as follows:

Difference X = X_gs_ − X_4D-JM_; difference Y = Y_gs_ − Y_4D-JM_; difference Z = Z_gs_ − Z_4D-JM_

Abbreviations are the same as described above.

## 3. Results and Further Data Exploration

**Figure 4:**
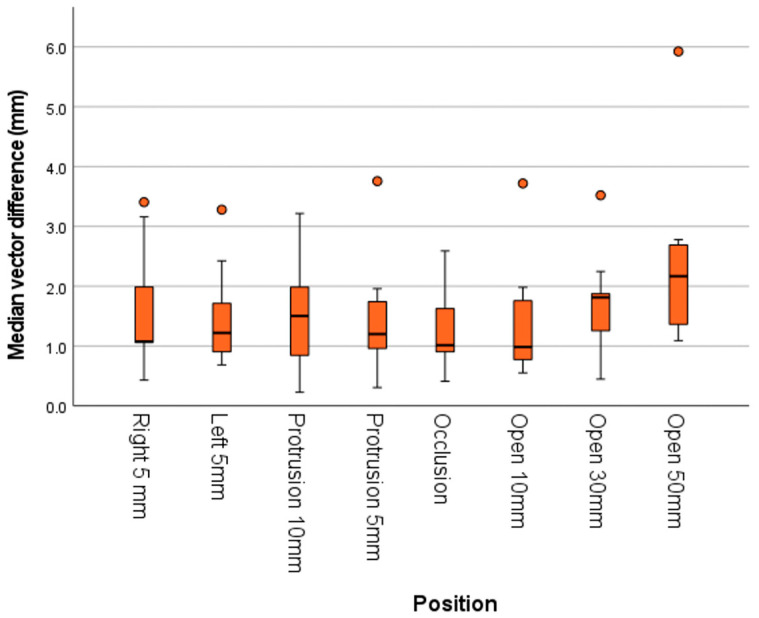
Box and whisker plots of the mandibular vector differences for different mandibular positions. With an increase in the vertical dimension the median differences increase.

All ICCs were 0.96 or higher (Table 1). The percentage of vector differences falling within 0.6 mm was 10% for the mandible and 90% for the maxilla (Table 2). All medians of the mandibular vector differences for the different wafer positions were larger than 0.6 mm (Figure 4). With a larger vertical opening, a larger variation in mandibular vector differences was found. For the position “Open 50 mm” an outlier was found; vector difference 5.9 mm. All vector differences of the maxilla fell within 1.2 mm of the gold standard. The mean vector difference of the maxilla was 0.3 mm (95% confidence interval: 0.3, 0.4) and differed significantly from 0 (t(71) = 15.006, *p* < 0.001).

**Figure 5:**
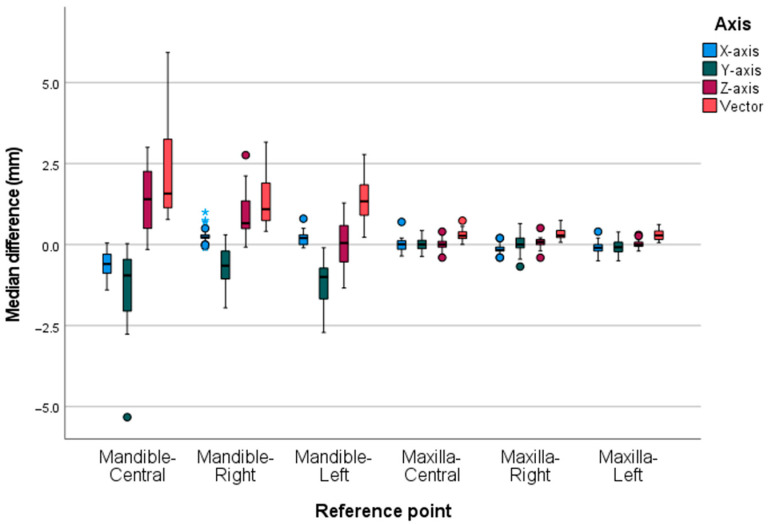
The box and whisker plot illustrates the median differences of the different reference points subdivided by the axes. The range of differences between the gold standard and the 4D-JM group were substantially larger for the mandible than for the maxilla. Within the mandible the range of differences of the point measurement of the Mandible–Central was largest. The far outliers (>1.5 IQR) of Mandible-Right are depicted as blue stars.

**Figure A1:**
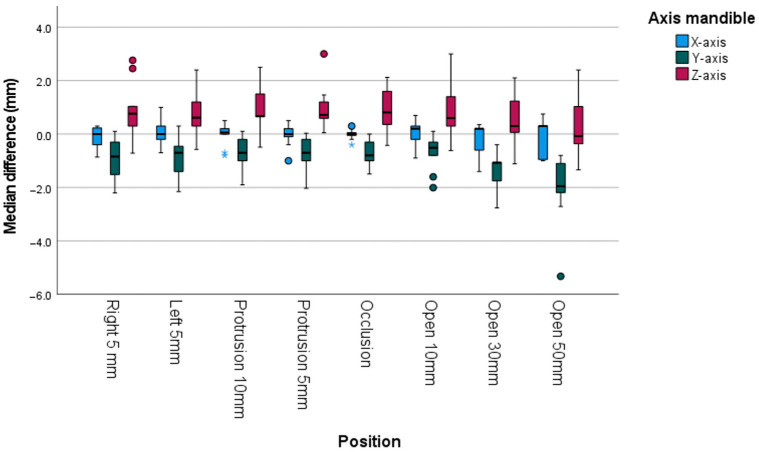
Box and whisker plot of median differences in the different mandibular positions divided by the axes. With an increase in vertical dimension a larger median difference occurs. Differences on the *x* axis show the least variation and are closely centered around zero. On the *y* axis, the 4D-JM overestimates distances and, on the *z* axis, the distances are underestimated. The far outliers (>1.5 IQR) of position Left 5 mm and Occlusion are depicted as blue stars.

The differences between the gold standard and 4D-JM group showed a substantially larger range for the mandible than for the maxilla (Figure 5). Among the mandibular reference points, the reference point Mandible–Central had the largest median difference. The smallest differences of the mandible were observed on the *x* axis. On the *y* axis, the distances were generally overestimated by the 4D-JM, while on the *z* axis the distances were generally underestimated (Appendix A, Figure A1). All absolute differences of the jaw, reference points and mandibular positions are presented in Appendix A, Table A1.

In the position occlusion, differences were found between the gold standard and 4D-JM for the mandible. Therefore, the 3D models were carefully inspected again because, theoretically, no movement should occur because the mandibular position relative to the skulls had not changed. It became clear that a shift occurred in the mandible position after the 4D-JM segmentation process, when the software connected with the CBCT scanner to register the start position of the 4D-JM tracking device. A backward rotational movement of the mandible was observed. The maxilla did not move; therefore, no superimposition was needed. The maxilla only showed differences in position between the gold standard and 4D-JM when wafers were inserted, and the skull needed to be repositioned. The gold standard CBCT captured this movement of the maxilla but the 4D-JM 3D model appeared to remain in the original position and, therefore, superimposition was required. The mandibular shift was further explored by repeating the startup protocol 30 times, and vector differences were calculated again. Median vector differences were for Mandible–Central 1.4 mm (SD 0.1 mm), for Mandible–Right 0.6 mm (SD 0.1 mm) and for Mandible–Left 0.9 mm (SD 0.1 mm). Median *z* axis differences were for the Mandible–Central 1.4 mm (SD 0.1 mm), while Mandible–Right and Mandible–Left both were 0.4 mm (SD 0.1 mm).

## 4. Discussion

The percentage of vector differences falling within 0.6 mm was 10% for the mandible and 90% for the maxilla. Before conducting the study, the authors established a predefined limit of 0.6 mm based on their clinical experience, as there was no available literature data on this matter. Initially, it was anticipated that the segmented 3D models of the gold standard and 4D-JM would closely resemble each other. However, the 4D-JM 3D model is segmented by a region growing method as a compressed hollow 3D model. This segmentation process led to surface differences between the gold standard CBCT 3D model and the 4D-JM 3D model. Due to the insertion of the wafers, a difference in the position of the skulls occurred between the segmented gold standard and 4D-JM 3D models, and therefore both 3D models had to be superimposed. A perfect superimposition was not possible due to the aforementioned surface differences. This imperfect superimposition is reflected in the differences of the maxillary reference points (Figure 5). The superimposition error was explored by analyzing the vector differences of the maxilla which appeared to be significantly different from zero. However, it is important to note that the superimposition error can only partly account for the mandibular vector differences exceeding 0.6 mm.

The mandible and maxilla differed in their median differences (Figure 5). Additionally, the *x* axis had the smallest range of differences for the mandible. The reference point of the Mandible–Central showed the largest differences, which might be ascribed to the largest movement on opening and to the reproducible startup shift of the segmented 4D-JM 3D model explained above. This shift is also illustrated in Figure 4 for the occlusion position (i.e., unaltered mandible position). This position showed the smallest median difference of the mandibular vectors. However, this difference is negligibly small compared to the difference occurring in the positions Open 10 mm, Protrusion 5 mm, Right 5 mm and Left 5 mm.

The start position recommended by the manufacturer is the teeth in maximum intercuspation, which can be reproducible in patients with a distinct interdigitation. However, in the dry skulls, this start position was difficult to attain because of the absence of a distinct interdigitation. While this difficulty might have slightly altered the start position between measurements in the different positions, it cannot explain the startup shift because the mandible had not been placed in another position.

The range of differences of the mandible was the smallest for the *x* axis, possibly due to the position of the two cameras, which are obliquely in front of the skull. The larger differences for the *y* axis and *z* axis might be caused by using only two cameras situated at the same height. With two cameras placed in different positions on the *x* axis, the 4D-JM might have difficulty computing the *y* axis and *z* axis coordinates of the reflective spheres by triangulation.

On the *y* axis of the mandible differences were generally overestimated (Figure 5 and Appendix A, Figure A1), while on the *z* axis the mandible differences were generally underestimated. Differences increased with an increasing vertical dimension or protrusion (Figure 4 and Appendix A, Figure A1). Two skulls showed comparable measurements, but the third skull showed larger differences for inexplicable reasons (findings can be requested from the corresponding author).

The use of 4D CT for tracking the mandibular and condylar movements has also been reported [8,9]. Moreover, 4D CT captures multiple CT images within a timeframe and can register the functional movements of the mandible. Huys et al. [8] compared 4D CT to a phantom device equipped with a controlled motor that allowed six degrees of freedom. For five repetitions, the average 95% confidence interval for the *x* axis was 0.4 mm, for the *y* axis 0.6 mm and for the *z* axis 0.2 mm. In our study, the median mandibular difference for the *x* axis was 0.1 mm [−0.3; 0.3], for the *y* axis −0.9 mm [−1.6; −0.4], and for the *z* axis 0.6 mm [0.1; 1.4]. A notable issue with 4D CT is the radiation exposure resulting from the longer exposure time. The effective dose of a 4D CT scan is 1.3 mSv, whereas the mean effective dose of a CBCT scan with large field of view is 0.212 mSv [10]. However, the longer exposure time of 4D CT imaging might be advantageous for capturing high occlusal loading, as occlusal loading can temporarily bend the jaws and condyles and affect tooth mobility [11]. On the other hand, an optoelectronic CBCT system treats the maxillomandibular complex as two rigid bodies and may therefore depict occlusal loading less accurately in vivo.

The SICAT JMT+ system, an ultrasound-based CBCT system, has recently been used to investigate the functional movements of the TMJs [4,5]. He et al. [3] explored the accuracy of the SICAT JMT+ for different jaw movements, including opening, protrusion, right and left lateral movements, over three consecutive days in a single patient [3]. The differences observed between the days ranged from 0.4 mm to 2.8 mm, but it is unclear whether these variances were due to differences in the patient’s jaw movements or measurement errors of the SICAT JMT+ system. The condylar position simulated with the SICAT JMT+ coincided with the condylar positions on a CBCT scan (the gold standard) [3].

In 2008, Terajima et al. [12] introduced an experimental optoelectronic system (TRI-MET, Tokyo, Japan), which allowed the tracking of mandibular movement with a CT scan. The 3D position of six light-emitting diodes (LEDs) attached to a mandibular and maxillary facebow were tracked in a single patient. The authors reported that the system provided precise simulations of mandibular movement, but they did not report any validation of their measurements.

An experimental optoelectronic system for registration of mandibular movements was analyzed for its validity by tracking a bar with two markers on it with a fixed distance of 50 mm. The bar moved up and down. The mean difference between the system and the true value of 50 mm was 0.4 mm (SD 0.3 mm, range −1 to 1.1) [13]. These findings suggest a good validity, but no CBCT scans were used to verify the measurement in patients. In our study, the median mandibular difference for the *z* axis was 0.6 mm [0.1; 1.4].

In a laboratory study, an optoelectronic and an electromagnetic tracking system were tested for validity using CT scanning as the gold standard [14]. Two plastic blocks were placed in a precision device which controlled translations and rotation between the blocks. The mean measurement error of the optoelectronic system for translation was 0.03 mm (SD 0.1 mm) and for curvilinear distances (length of path from start to end position) 0.4 mm (SD 0.2 mm). In our study, we used 3D translations and measured the 3D distances between the gold standard and the 4D-JM. Pinheiro et al. [13] and Baltali et al. [14] both used 2D-translation paths and 2D measurements to measure the accuracy of their optoelectronic tracking system. A vector is mathematically always equal or larger than the largest differences of the *x*, *y* and *z* axis. Therefore, analyzing the accuracy of an optoelectronic tracking system with solely 2D-measurements might overestimate accuracy of the system.

The 4D-JM system was applied to investigate a patient specific rotation center for total temporomandibular joint replacements [6]. In that study, the validity of the entire workflow, including 4D recordings was analyzed using a phantom. The mean Euclidean error (vector difference) of start and end coordinates of the rotation axis was 0.8 mm, but no standard deviations were reported; thus, the variation of these errors is unclear.

The smallest detectable difference (SDD) of mouth opening for being successful in painfully restricted temporomandibular joint patients ranges between 6 and 9 mm [15]. In juvenile idiopathic arthritis patients, the SDD is 4.9 mm for mouth opening, 2.4 mm for laterotrusion and 2.8 mm for protrusion [16]. In the current study, the accuracy of the 4D-JM is much better than these values, but before the 4D-JM can be applied clinically, validity should be tested in patients.

The accuracy of the 4D-JM could potentially be improved by replacing the region growing segmentation tool with a different segmentation method that has a lesser impact on the quality of the 3D model. Additionally, enhancing the tracking of the 4D-JM reflective spheres could be achieved by using four cameras instead of two [17]. Furthermore, replacing the used passive markers with active markers (e.g., infrared LED) could also be considered. A possible solution for a reproducible start position could be utilizing a small individualized radiolucent wafer. This wafer would not only provide a consistent starting point but would also create freeway space, which could enhance the segmentation time required to separate the mandible and maxilla. The potential of the 4D-JM is promising, provided that its accuracy can be improved and its clinical validation proves successful.

This study investigated 3D distances using jaw motion tracking devices capable of tracking 3D-curvilinear paths. A limitation of this study is the use of dried skulls, making it difficult to capture these paths due to the control CBCT scans that needed to be taken. Future research could consider 3D path-capturing with a motorized linear and rotary stage mounted to the skull, positioned outside the field of view of the CBCT scanner. A potential contributing factor to the inaccuracy of the 4D-JM in the current study set-up could be the difficulty in reproducing the start position of the segmentation process. This might be improved by using a small individualized radiolucent wafer. Additionally, the sample size of this explorative study was small, making it difficult to explain the cause of the differences between the three skulls.

## 5. Conclusions

The aim of the study was to explore the validity of the 4D-JM for different mandibular positions. The mandibular vector differences of the 4D-JM did not meet the predefined criterion of 0.6 mm. Potential factors contributing to the measurement error are the region growing segmentation method of the 4D-JM, the startup shift of the mandible, and the lack of occlusal interdigitation of the skulls. While the 4D-JM optoelectronic integrated CBCT scanner appears to be a promising jaw motion tracking device, in our study set-up, it was not accurate enough to meet the authors’ predefined standards.

## Figures and Tables

**Table 1:** ICC Absolute agreement.

Observer	Single Measures	95% CI (Lower Border)	95% CI (Upper Border)
HH1-HH2	0.973	0.967	0.977
HH1-AD1	0.963	0.955	0.969
HH2-AD2	0.970	0.964	0.975
AD1-AD2	0.964	0.957	0.970

ICC: Intraclass correlation coefficient (two-way mixed effects model, absolute agreement, single measures); CI: Confidence Interval; HH1: first observer first measurements; HH2: first observer second measurements; AD1: second observer first measurements; AD2: second observer second measurements.

**Table 2:** Percentage of vector differences less than 0.6, 1.2, 1.8 and 2.4 mm.

	Vector Differences Less Than:			
	0.6 mm	1.2 mm	1.8 mm	2.4 mm
Mandible	10%	42%	65%	83%
Maxilla	90%	100%	100%	100%

## Data Availability

Data presented in this study can be found in Figshare: https://doi.org/10.6084/m9.figshare.22801706.v1.

## Appendix A

**Table A1:** Absolute *x*, *y* and *z* axis differences and vector differences between gold standard and 4D-JM reference points.

Absolute Differences	*X* Axis	*Y* Axis	*Z* Axis	Vector
Mandible, median [IQR] (mm)	0.3 [0.2; 0.6]	0.9 [0.4; 1.6]	0.7 [0.4; 1.4]	1.3 [1.0; 2.0]
Maxilla, median [IQR] (mm)	0.2 [0.1; 0.2]	0.1 [0.0; 0.3]	0.1 [0.0; 0.2]	0.3 [0.2; 0.4]
Reference points ^a^, median [IQR] (mm)				
Mandible–Central	0.6 [0.3; 0.9]	1.0 [0.5; 2.0]	1.4 [0.5; 2.3]	1.6 [1.1; 3.2]
Mandible–Right	0.2 [0.2; 0.3]	0.6 [0.3; 1.1]	0.7 [0.5; 1.3]	1.1 [0.7; 1.9]
Mandible–Left	0.2 [0.1; 0.3]	1.0 [0.7; 1.7]	0.6 [0.2; 0.7]	1.3 [0.9; 1.8]
Maxilla–Central	0.1 [0.1; 0.2]	0.1 [0.0; 0.3]	0.1 [0.0; 0.2]	0.3 [0.2; 0.4]
Maxilla–Right	0.2 [0.1; 0.2]	0.1 [0.0; 0.3]	0.1 [0.1; 0.2]	0.3 [0.2; 0.4]
Maxilla–Left	0.1 [0.0; 0.2]	0.1 [0.1; 0.3]	0.1 [0.0; 0.1]	0.3 [0.2; 0.4]
Mandibular positions ^b^, median [IQR] (mm)				
Right 5 mm	0.3 [0.2; 0.4]	0.8 [0.3; 1.5]	0.8 [0.5; 1.0]	1.1 [1.1; 2.0]
Left 5 mm	0.3 [0.2; 0.7]	0.7 [0.5; 1.4]	0.6 [0.3; 1.2]	1.2 [0.9; 1.7]
Protrusion 10 mm	0.2 [0.1; 0.5]	0.7 [0.2; 1.0]	0.7 [0.6; 1.5]	1.5 [0.8; 2.0]
Protrusion 5 mm	0.2 [0.1; 0.4]	0.7 [0.2; 1.0]	0.7 [0.6; 1.2]	1.2 [1.0; 1.7]
Occlusion	0.1 [0.0; 0.2]	0.8 [0.3; 1.0]	0.8 [0.4; 1.6]	1.0 [0.9; 1.6]
Open 10 mm	0.3 [0.2; 0.5]	0.5 [0.3; 0.8]	0.6 [0.5; 1.4]	1.0 [0.8; 1.8]
Open 30 mm	0.3 [0.2; 0.6]	1.1 [1.1; 1.8]	0.6 [0.2; 1.2]	1.8 [1.3; 1.9]
Open 50 mm	0.5 [0.3; 1.0]	2.0 [1.1; 2.2]	0.7 [0.4; 1.3]	2.2 [1.4; 2.7]

Interpretation colors: Absolute median differences between gold standard and 4D-JM reference points: 0.0–0.6 mm (green); 0.7–1.2 mm (orange); 1.3–2.4 mm (red). Note: (^a^) Reference points include all mandibular positions; (^b^) mandibular positions include the reference points: Mandible–Central, Mandible–Right and Mandible–Left.
